# Supplementary material for: Are carbon emissions trading and green financial instruments synergistic? -Comprehensive quantitative research based on content analysis
Source: PLoS One. 2024 Mar 7;19(3):e0298601. doi: 10.1371/journal.pone.0298601 (PMC10919601; doi:10.1371/journal.pone.0298601)
Supplement: S1 Data — (ZIP) [file pone.0298601.s001.zip › Data/地级市Z_F工作报告.zip!/║┌┴·╜¡/╝a╬≈╩╨/└┤╫╘╣1⁄2╓┌║┼í╛┬φ┐╦╩2╛▌═°í┐_2015.docx]

各位代表：

现在，我代表市政府向大会作工作报告，请予审议。

一、2014年工作回顾

2014年是鸡西发展进程中极不平凡的一年。面对经济下行的巨大压力，在省委、省政府和市委的坚强领导下，在市人大、市政协的监督支持下，全市人民团结一心、迎难而上、奋发有为，经济发展呈现出企稳向好的态势。全市地区生产总值实现540.9亿元，增长1%；规模以上工业增加值70.7亿元，下降11.3%；固定资产投资207.8亿元，下降11.2%；社会消费品零售总额204.3亿元，增长10.8%；外贸进出口总额8.2亿美元，下降31.5%；公共财政预算收入33.7亿元，增长0.8%；城镇居民人均可支配收入19375元，增长4.7%；农村居民人均可支配收入13449元，增长12.2%。

——项目建设亮点纷呈。启动实施投资5000万元以上产业项目106项，当年完成投资72.6亿元。总投资25亿元的北汽集团锂电负极材料及石墨深加工项目，开创了单体产业项目投资规模最大的先河。投资50亿元的汇源生态农业综合开发、6.3亿元的青岛啤酒异地扩建、5亿元的娃哈哈饮料二期、4.8亿元的珍宝岛药业(鸡西)二期等一批项目开工建设，全市项目形成了体量大、结构好、质量优的新格局。

——对上争取成效显著。共争取到位资金69亿元，增长12.2%，创历史新高。争取国开行支持棚户区改造授信额度37.8亿元；鸡冠城区老工业区和滴道独立工矿区改造搬迁获得国家三年政策支持；《兴凯湖流域水环境保护综合规划》通过国家发改委评审；兴凯湖良好湖泊保护项目国家4.67亿元资金已拨付到位；虎列国际铁路、虎林支线机场等基础设施项目纳入省规划。

——产业转型全线提速。农业“两个转变”成效初显。粮食喜获丰收，总产量达61.3亿斤。绿色食品种植基地监测面积达到495万亩，新增国家级农业产业化重点龙头企业1户，省级龙头企业3户。农产品创驰名商标2个、著名商标14个、地理标志证明商标3个。畜牧业发展强劲，新建万头奶牛基地1处，千头牛场5处，万头猪场7处。畜牧业总产值实现47亿元，同比增长9.1%。工业“双轮驱动”转速加快。“两黑一绿”产业快速发展，鸡西煤化工园区A区(鸡东)3平方公里核心区开工建设。北汽、贝特瑞、瑞诺恒等一批石墨深加工项目顺利实施，我市被中国矿业联合会授予“中国石墨之都”称号，成为全省首家中国矿业名城。食品医药产业发展迅速，正在成为接续替代产业和骨干支撑产业。企业裂变升级稳步推进，65个裂变项目开工建设。三产“旅贸牵动”活力增强。开工建设了虎林月亮岛中俄风情休闲娱乐城等20余个旅游项目。新开通4条跨境游线路，兴凯湖风景区成为省重点推介五大景区之一。开工建设了密山中俄边民互市贸易区，举办了中俄(鸡西)义乌小商品博览会等展会。全年金融机构存款余额855.7亿元，贷款累放335.9亿元。

——重点改革稳步推进。现代农业综合配套改革全面推开。完成卫生计生机构合并，工商、质监职能接收工作。行政审批事项由409项精简到314项。启动了市级部门综合预算改革。我市被列为全省购买公共服务工作试点市。完成龙煤集团供水交接工作。实施了2线等公车公营改革。

——城市功能日趋完善。改扩建鸡兴西路、康新路等22条城市街路，实现了新老城区互联互通。实施南星街景观带和主要街路、游园广场绿化工程，新增绿地101公顷。改造供水管线9.6公里、供热管网66公里，新建换热站10座。投入2660万元购置的清运雪设备投入使用，机械化作业能力明显提高。

——民生实事质优量足。全年财政投入民生资金79.8亿元，占财政总支出58.6%，11件实事全部完成节点任务。投资23.3亿元的城市供水工程开工建设；穆棱河大桥、团结立交桥、左家立交桥、滴道公铁立交桥实现了当年设计、当年施工、当年通车，创造了鸡西速度；城市森林公园顺利推进，东山桥公园建成开放。百条背街巷道全面竣工，改造弃管楼263栋，创建标准化社区12个。此外，解决了3300多户居民房照办理和4700多户居民超期回迁等群众反映强烈的热点难点问题。

——社会事业蓬勃发展。调整完善创业扶持政策，建立了大学生创业示范基地，发放创业扶持资金4520万元。新建校舍2.3万平方米，发放助学资金3114万元，资助贫困学生3.6万人。新农合人均筹资标准提高到390元，参合率达到99.8%。城镇居民低保标准提高到450元/月，农村居民提高到2800元/年，均高出省定标准。规范提高机关事业单位工作人员及离退休人员津贴补贴标准，从2014年1月起，在职人员人均月增资215元，离退休人员人均月增资383元。新建33个村级文化活动室，17个社区文化活动中心。举办了四国女篮挑战赛和环兴凯湖国际马拉松公路接力赛，在省第十三届运动会上获金牌96枚。社会治安持续好转，刑事案件发案量同比下降16.3%。及时妥善解决了一批重点领域信访突出问题，全市信访形势总体平稳。

——行政效能明显提升。进一步完善了政府工作规则、重大决策规则等制度。在全省率先开通网上政务服务中心，实现了审批提速。在市政府网站公开了财政预决算和“三公”经费等方面情况，提高了工作透明度。人大代表、政协委员的415件议案、建议和提案全部办理。结合党的群众路线教育实践活动，集中治理“慵懒散漫浮”问题，政府机关工作效率和服务意识明显提升。

各位代表，回顾过去的一年，我们深感成绩来之不易，这主要得益于省委、省政府和市委的正确领导，得益于市人大、市政协和社会各界的鼎力支持，得益于全市人民的团结奋斗。在此，我代表市政府向全市广大干部群众，向所有关心和支持鸡西发展的同志们、朋友们，表示衷心的感谢，并致以崇高的敬意！

当前，我市经济社会发展还存在不少困难和问题。经济下行压力大，稳增长的内生动力不足；“一煤独大”的产业矛盾突出，接续替代产业规模小；受煤炭市场持续低迷影响，涉煤企业生产经营尤为艰难；县(市)区大项目、好项目、财源项目少；煤矿安全生产形势严峻；就业再就业、采煤沉陷区治理等民生任务重；发展环境有待进一步优化，政府公务人员推动发展的本领和劲头尚需提升，等等。对此，我们将采取有力措施，认真加以解决。

二、2015年工作安排

2015年，市政府工作的总体思路是：深入贯彻落实“四个全面”战略布局，牢牢把握省实施“五大规划”和建设“中蒙俄经济走廊”黑龙江陆海丝绸之路经济带的战略机遇，按照市委十二届六次全会的战略部署，以稳中求进为总基调，以转型发展为主线，以改革创新为动力，坚持转方式调结构并重、提质量增效益并重、惠民生促和谐并重，坚定不移推进“三大战略”(两个转变、双轮驱动、旅贸牵动)，做强做优“四大主导产业”(两黑一绿一药)，推动全市经济社会持续健康发展，全力开创鸡西“转型发展兴市富民”新局面。

经济社会发展的主要预期目标是：地区生产总值增长3%，规模以上工业增加值增长1%，固定资产投资增长1%，公共财政预算收入增长2%，社会消费品零售总额增长10%，外贸进出口总额增长5%，城镇居民人均可支配收入增长5%，农村居民人均可支配收入增长10%。节能减排指标达到省核定标准。

为实现上述目标，重点抓好七个方面工作。

(一)坚定不移上项目，进一步构筑经济增长支撑力

项目建设是加快发展的引擎、结构调整的抓手、兴市富民的基石，坚持把全部精力向项目集聚、所有力量向项目投放，倾力建设一批打基础、促转型、增后劲、利长远的大项目、好项目。

加大项目建设力度。启动实施投资5000万元以上产业项目104个，当年完成投资113.2亿元。大力推进投资6亿元的汇源40万吨生鲜乳、3亿元的中航重工秸秆板材加工等71个新开工项目。北汽集团3万吨锂离子电池负极材料项目竣工投产，完成青岛啤酒一期20万千升改扩建工程。珍宝岛药业(鸡西)二期、福娃稻米食品深加工等项目实现竣工。

提升招商引资实效。突出重点招商，瞄准央企国企、大型民企的投资方向，大力引进战略投资者。突出专题招商，围绕“两黑一绿一药”，精心组织各类招商活动，积极引进一批行业领军企业。突出以商招商，力争引进一批新的投资主体。加强项目跟踪和服务，提升合同履约率、资金到位率、项目开工率。

增强园区承载功能。有序推进鸡西煤化工园区A区3平方公里核心区的道路、给排水、污水处理厂等基础设施建设；完成B区(梨树工业谷)四、六区土地征收，腾空土地34公顷。加快石墨产业园区项目入驻步伐，促进产业配套发展、集群发展、高端发展。做好鸡西(鸡冠)产业园区土地收储，加速扩模升级。深入落实飞地项目利益分享机制。

(二)坚定不移抓转型，进一步增添工业发展原动力

深入实施“双轮驱动”战略，坚持扩张存量与引进增量并重、打造龙头企业与培育产业集群并举，促进工业经济提质增效。

打造四大产业集群。围绕煤炭、石墨、绿色食品、医药四大产业，着力构建特色鲜明、结构优化、体系完整的立市产业群。做实煤化工产业，围绕煤制油、煤制烯烃，深度谋划50万吨中低温煤焦油加氢、400万吨低阶煤提质项目，完善项目前期，落实项目主体。开工建设龙昌硅藻土开发等项目，推动天和焦化、天诚镁合金联产项目尽快投产。做精石墨加工产业，围绕载能材料、密封材料等七大产业链，加快建设贝特瑞锂电负极材料、活性炭等一批深加工项目，完成国家石墨产品质量监督检验中心验收，发挥国家级石墨产业技术创新战略联盟作用，叫响“中国石墨之都”城市名片。做大绿色食品产业，发挥我市农副产品优势，坚持以工业化理念，推进粮食产品、畜牧产品和山特产品深加工，积极开发功能食品、休闲食品、保健食品，重点推进中豆植物蛋白乳、钲祥北虫草等一批投资超亿元项目，打造龙江绿色食品强市。做强医药产业，支持珍宝岛药业加快发展，推进乌苏里江制药香丹注射液、富春堂阿胶制品、新医圣制药蒲参盆腔泰胶囊等项目建设，盘活完达山药业。力争用2—3年时间，实现医药产业产值倍增，占工业总产值比重15%以上。

推进企业裂变升级。以打造“百亿航母、十亿板块、亿元群体”为目标，实施45个企业裂变升级项目。重点抓好完达山1万吨白酒生产线、珍宝岛药业(虎林)8000万支冻干粉针综合制剂等扩能改造项目；推进金宇1.7万吨石墨深加工制品、新科年产120万吨洁净配煤厂等改造升级项目。同时，全力支持鸡西矿业公司、沈煤集团鸡西盛隆公司解困发展，深入开展与农垦、森工合作，推动区域经济联动发展。

(三)坚定不移促转变，进一步提高现代农业竞争力

以“两大平原”现代农业综合配套改革试验为引领，深入推进“两个战略转变”，促进农业增效、农民增收。

加快发展现代畜牧业。以打造全省重要的优质奶源生产基地和畜产品加工基地为目标，推动畜牧养殖、加工销售、冷链物流为一体的全产业链发展。推进标准化建设，坚持畜禽养殖“三退三进”、“四统一”，新建3处万头猪场、2处千头牛场和10处市级标准化规模养殖场，建设万亩优质饲草基地。推进规模化养殖，重点建设汇源10万头、娃哈哈1万头、天翔2000头奶牛等一批大型现代牧场。推进产业化发展，加快娃哈哈、汇源等龙头企业建设，引进一批畜牧精深加工企业，大力发展乳制品、肉制品精深加工，壮大畜牧加工企业群体。

深入推进绿色品牌农业。打造一流基地，按照省建设亿亩生态高产标准农田部署，编制规划，适时启动实施。推广测土配方施肥、森柒复合酶等先进技术和产品，严格质量监管，确保我市绿色食品立得住、叫得响、推得开。全年绿色食品种植基地监测面积达到500万亩。积极培育农民合作社、家庭农场等新型农业经营主体，完善社会化服务体系，稳步推进规模经营。发展精优特农业，因地制宜扩大棚室蔬菜、白瓜、西香瓜、万寿菊等作物种植面积，推广桑蚕、河蟹等特色养殖。实施品牌战略，组建鸡西知名(商标)品牌战略联盟，整合资源，统一策划、统一包装、统一宣传。推动我市大米、蜂蜜等绿色品牌进入省百家旗舰店、千家连锁店、万家主流超市，积极组织企业参加全国绿色食品博览会，提高我市绿色食品知名度和市场占有率。

培育壮大北药产业基地。坚持种加销一体化、医药产业配套化，突出合作社、大户带动，进一步扩大刺五加、北虫草、桔梗、人参等药材种植面积，北药基地达5万亩。加强产学研结合，开发一批新型北药产品、保健食品和功能饮品，力争引进一批大企业、大项目，引领北药产业化发展。加快梨树山产品(药材)批发大市场建设，培育经纪人队伍，发展北药物流。

(四)坚定不移兴三产，进一步激发消费市场新活力

深入实施“旅贸牵动”战略，提高第三产业发展水平和比重，打造新的经济增长点。

做强旅游业。启动实施《鸡西旅游总体规划》，做好“延点、延线、延时”三篇文章。围绕“一核、一带、三区、五基地”，深度挖掘重点景区历史文化内涵，打造主题鲜明、特色突出的四季旅游项目。精心策划旅游产品，高水平举办观鸟节、夏季露营大会、冬捕冬钓、冰雪汽车拉力赛、全国城市公路接力赛等活动。加快虎林月亮岛中俄风情休闲娱乐城、凤凰山水上乐园等重点旅游项目建设，争取虎头珍宝岛景区晋升国家5A级景区。整合域内旅游资源，对接周边知名景区，加强与国内百强旅行社合作，精心推出休闲避暑、湿地观光、红色文化、跨境风情等一批旅游线路。深入研究旅游半径，面向航空目的地、友好城市，借助携程、途牛等知名网站和传统媒体，加强线上线下宣传营销，扩大鸡西旅游知名度和影响力。

活跃商贸物流业。大力发展电子商务，出台促进电子商务产业发展的实施意见和扶持政策，加快建设中小商贸流通企业电子商务平台和数据中心。积极引进阿里巴巴等知名电商企业和互联网公司，加快搭建绿色农产品、小商品、跨境电子商务、智慧旅游等电子商务平台，吸引中小企业和商户入驻，重点扶持100家电商企业发展壮大。提升物流业层次，加快市交通智能服务管理平台建设，构建公铁联运、集装箱运输、同城配送物流体系，突出抓好北京翰沃集团物流等重点项目建设，积极发展农产品冷链、医药配送等物流业态，打造区域物流中心。发展外向型经济，抓住省建设“中蒙俄经济走廊”黑龙江陆海丝绸之路经济带契机，积极推进虎列国际铁路、牡佳快速铁路环线鸡西段等项目建设。坚持出口抓加工、进口抓落地，加强虎林、密山进出口加工园区建设，培育壮大外贸加工企业群体，推动外贸产业转型升级。

繁荣文化产业。提炼升华肃慎、抗联、北大荒等文化内涵，打造富有地域特色的城市文化。加强版画之乡、剪纸之乡、摄影之乡三大文化品牌基地建设，推动文化与旅游、金融、科技等产业融合发展，培育一批特色鲜明、创新能力强的骨干文化企业，提升文化产业竞争力和影响力。

壮大现代服务业。积极引进外埠金融保险机构，做强服务主体。推进农村融资担保服务体系建设，鼓励支持企业直接债务融资。严厉打击恶意逃废金融债务等行为，进一步优化金融生态环境。积极发展健康养老、影视传媒、教育培训等服务业。办好中俄(鸡西)义乌小商品博览会等展会。

(五)坚定不移搞建设，进一步增强宜居家园吸引力

按照“一河居中、两岸同兴，东跨南进、西拓北连”的发展思路，进一步优化城市布局，全面推进城乡建设。

建设节能型城市。倡导节能城市建设理念，完成50万平方米既有建筑节能改造，加快推进市垃圾焚烧发电项目建设。改造市中心区31公里老旧供热管网，实施中心区4000户居民全天供水分户改造，完成冶金街排水和永昌西路雨水管线建设，推进西部排水工程。

建设生态型城市。谋划推进穆棱河、“两沟一河”水系建设，加快穆棱河风光带、鸡冠山森林公园建设。编制城市色彩规划，确定城市主色调，打造富有地域特色的城市风格。启动实施《兴凯湖流域水环境保护综合规划》，推进兴凯湖、穆棱河等防治项目建设；加强秸秆综合利用、大气污染防治和主要污染物减排，提升空气质量；加大退耕还草、还湿力度，治理水土流失4万亩，植树造林3.3万亩。结合采煤沉陷区棚户区改造，逐步推进矿区道路、供水、供热、供气、排水，垃圾转运等基础设施建设，进一步改善矿区人民群众生产生活环境。

建设智慧型城市。推进智慧鸡西建设，打造以政务、产业、民生为核心的智慧城市管理平台，力争进入全国智慧型城市试点。加强城市执法、市政环卫、园林物业、城市交通等智能化管理，建立城市管理协同和快速反应机制。开展文明城市创建活动，形成全民共建美好家园的浓郁氛围。建设宜居型美丽乡村。启动实施新一轮美丽乡村建设三年行动计划。加强农村道路、饮水安全等基础设施建设，推进村屯绿化、美化、净化。积极推进特色乡村建设，打造一批农业观光村、休闲度假村、民族风情村、特色旅游村，让农村成为令人向往的美丽家园。

(六)坚定不移惠民生，进一步构建和谐社会

保障力把保障和改善民生作为一切工作的出发点和落脚点，持续加大民生投入，切实解决好关系群众切身利益的大事难题，重点办好15件民生实事。(1)实施创业“十百千万”工程，打造10个创业示范基地，选树100个创业典型，开发1000个创业项目，新增1万个创业实体，大力扶持中小微企业，营造“大众创业、万众创新”的良好局面。(2)全力推进东升花园、森林名苑2个政府融资主导建设项目，新建百万平方米安置住宅，让万户居民“出棚进楼”，实现安居梦。(3)加大采煤沉陷区棚户区改造力度，异地搬迁安置工程两年全部开工，三年建设完成，当年安置22979户，解群众之急、暖百姓之心。(4)投资8000万元，改造弃管楼200栋。(5)全面贯彻落实《省物业管理条例》，继续开展“物业管理提升年”活动，切实提高物业管理水平，让百姓生活得更舒心。(6)改造背街巷道117条，切实解决群众行路难问题。(7)投资4000万元，开工建设市公路客运枢纽。(8)规范停车场秩序，取缔乱收费行为。(9)投资1.6亿元，建设穆棱河众兴跨河新桥。(10)投资2600万元，开工建设鸡图公路平岗至梨树段13公里大修改造工程。(11)开工建设动植物园二期，完成城市森林公园建设，努力建设更多的城市绿洲。(12)开工建设永久燃气供气场站，整合全市供气管网，让2万户市民先期用上优质高效的清洁能源。(13)投资4020万元，解决5.9万农村人口的饮水安全问题。(14)新建校舍1.4万平方米，资助学生3.5万人，投资9800万元完成教育信息化“三通两平台”工程建设，建立完善现代职业教育体系，努力办好人民满意教育。(15)建立市企业登记服务中心，推进“四证合一”，为企业提供便利高效服务。

(七)坚定不移保安全，进一步强化社会稳定

控制力坚决把安全生产作为压倒一切的硬任务，用最严格的监管、最严厉的处罚、最严肃的问责，推动安全生产形势稳步好转。

全力抓好煤矿安全生产。坚持“党政同责、一岗双责”，按照“五级五覆盖”要求，全面落实安全生产责任，打好停产整顿、整合关闭、隐患治理、鸡东煤矿安全全国重点县四个攻坚战。稳步推进地方煤矿整治整合关闭工作，鼓励支持龙煤、沈煤托管地方煤矿，完成64处煤矿关闭任务。加强瓦斯、水患治理，严抓严管，严防严控，坚决避免重特大事故发生。强化地方煤矿安全监管，采取“四不两直”方式，加大巡查检查密度，从严“打非治违”，从重打击超层越界、隐蔽工程等违法违规行为。严格执行安全生产一票否决和责任追究制，从严查处违法违纪人员。

全力推进地面安全。切实抓好非煤矿山、危险化学品、烟花爆竹等行业安全监管，加强道路交通、消防森防、食品药品、建筑施工等重点领域和商场、学校等人员密集场所的安全生产工作。完成22座非煤矿山关闭任务。

全力维护社会稳定。落实信访包案责任制，积极化解信访积案，切实解决好群众反映强烈的热点难点问题。加强公安“四项建设”，完成市中心区技防二期、交通指挥中心和城乡技防建设。推进警力下沉，强化街面屯警巡逻反恐处突，深入开展严打专项整治活动，始终保持对违法犯罪行为的高压态势。完善应急管理机制，有效应对各类突发事件，确保人民群众生命财产安全。加强平安边防建设，维护边境安全稳定。

三、着力推进改革和政府自身建设

坚持以法治思维推进政府工作，以政府改革带动重点领域改革，加快建设法治政府、廉洁政府和服务型政府，增强政府执行力和公信力。

坚持以担当为荣。大力弘扬担当精神，建立健全激励机制，引导广大干部主动到招商引资最前沿去，到棚户区改造等工作难度大的地方去，到产业项目建设等急难险重任务第一线去，在一线推进工作，在现场解决问题。对不作为、乱作为、慢作为严查严处、“零容忍”，旗帜鲜明地支持担当者、保护担当者、褒奖担当者，使敢于担当成为政府各级干部的自觉追求。

坚持以实干为要。用心谋事，从全局和长远角度谋划一批大事要事，办成一批实事好事，办妥一批急事难事，着力解决好事关广大人民群众切身利益的就业、教育、医疗、社会保障等问题，使人民群众生活有新提高、环境有新改善、日子有新变化。真心干事，把真抓实干贯穿于政府工作全过程，说了就办，定了就干，凡是做出的决定、出台的文件、召开的会议，全部建立督查落实机制，明确责任单位、责任人员和完成时限，做到言必行、行必果。对项目建设，实行市级领导包保责任制，对重点产业，成立专题推进组，专项推进，务求实效。

坚持以改革为先。继续推进行政审批制度改革，取消和下放一批行政审批事项，大力清理非行政许可审批，加快建立法无授权不可为的“权力清单”、法定职责必须为的“责任清单”。稳步推进国企改革，做好龙煤集团移交企业办社会接收工作。深入推进财税金融、社会保障、公务用车、公交改革、公共资源配置市场化等重点领域和关键环节改革。同时，以改革的精神、创新的理念，完成“十三五”规划编制。

坚持以法治为纲。推进“法治鸡西”建设，依法规范政府职权，把政府工作全部纳入法治轨道。主动接受人大法律监督和政协民主监督，认真办理人大代表建议和政协委员提案。健全依法决策机制，建立政府法律顾问、重大决策终身责任追究等制度。严格规范执法行为，重点治理野蛮执法、越权执法、以罚代管等行为。深入推进“六五”普法，加强法治文化建设，在全市形成办事依法、遇事找法、解决问题用法、化解矛盾靠法的浓郁氛围。

坚持以环境为重。开展“发展环境建设年”活动，集中开展突出问题专项整治行动，全面清理涉企不合理收费，严格规范中介机构服务行为，切实减轻企业负担。大力推进政务公开，及时发布政务信息。强化市企业投诉服务中心、网上政务中心和部门服务窗口职能，对招商项目实行“领办代办”制、首问负责制，专人负责，限时办结。健全问题快速处理机制，以铁的手腕严肃查处破坏经济发展环境的行为，为企业发展提供宽松环境。

坚持以廉洁为本。严格落实中央“八项规定”、省市“九项规定”精神，深入开展“三严三实”专题教育，坚持不懈纠正“四风”，进一步压缩会议规模和文件数量，严控“三公”经费。严格执行廉政准则，落实落靠“一岗双责”和“三重一大”制度。严格监督审计，强化对土地出让、产权交易、政府采购、工程招投标等重点领域和关键环节的监管，加强政府投资建设项目竣工结算审计。

各位代表，新起点孕育新希望，新常态面临新机遇，让我们在市委的正确领导下，万众一心，众志成城，开拓进取，奋发有为，为谱写鸡西“转型发展兴市富民”的新篇章而努力奋斗。
